# Ultralight, Strong and Renewable Hybrid Carbon Nanotubes Film for Oil-Water Emulsions Separation

**DOI:** 10.3390/membranes11010001

**Published:** 2020-12-22

**Authors:** Yamei Lu, Yingze Cao, Yi Jia, Chunai Dai, Pengfei Wang

**Affiliations:** 1 Department of Chemistry, School of Science Beijing Jiaotong University, Beijing 100044, China; 18126243@bjtu.edu.cn; 2 Qian Xuesen Laboratory of Space Technology, China Academy of Space Technology, Beijing 100094, China; caoyingze@qxslab.cn (Y.C.); jiayi@qxslab.cn (Y.J.)

**Keywords:** emulsion separation, hybrid CNTs film, ultralight film, combustion regeneration

## Abstract

A novel ultralight superhydrophobic-superoleophilic hybrid Carbon Nanotubes (CNTs) film with double-layer structures is fabricated by using vacuum filtration method. The CNTs film can separate various surfactant-stabilized water-in-oil emulsions with a separation efficiency higher than 99.3%. Moreover, the hybrid films can be regenerated through a simple and rapid combustion process within 2 s. In addition, the CNTs film still retains good hydrophobic properties under the conditions of physical abrasion, and strong acidic and alkaline solutions, which shows the excellent durability. The hybrid CNTs film is ultralight, stable, and easily stored and reused. The outstanding features of the obtained CNTs films we present here may find many important applications in various fields like oil purification and wastewater treatment.

## 1. Introduction

Nowadays, large amounts of oily wastewater have been discharged with the improvement of daily life and the development of industry [1]. Oil–water separation has been a worldwide challenge due to the tremendous threats caused by oil–water mixtures, which is harmful to the environmental as well to human health [2,3,4,5]. Generally, there are two kinds of oil–water mixture: stratified oil-water mixtures and emulsified oil–water mixtures [6]. Stratified oil–water mixtures containing larger dispersed droplets (>20 μm) are usually thermodynamically unstable and can be naturally separated over time, for a denser phase that gradually settles down and a lighter phase floats to the top. While the emulsified oil–water mixtures are relatively thermodynamically stable, micro/nano-scale emulsified droplets are generally stably dispersed in the continuous phase [7]. Due to the presence of the strong oil–water interface film and adsorbed interfacial active ingredients, the natural separation of the emulsified mixture relies on an impractically long timescale. In addition, the mixture generally becomes more difficult to separate as the size of the emulsified droplets decreases. Therefore, it is particularly important to develop reliable and performance-stable oil–water emulsion separation materials [8].

The conventional approaches to oil–water separation, such as gravity separation, centrifugation, electro-coalescence, and membrane separation have been widely used in water treatment [9,10,11,12,13]. However, these methods have quite a few drawbacks in practical use, like high cost, pollution, low separation efficiency, and so forth. The limitations of traditional methods have encouraged people to develop other advanced techniques for efficient and eco-friendly separation of oil–water mixtures. Recently, membrane materials with special wettability for oil–water separation have attracted substantial attention due to their advantages of simple operation, high separation efficiency and being friendly to environment [14,15,16,17]. Superhydrophobic/superoleophilic materials can be designed and fabricated by adjusting the surface structure and surface energy. The opposite wettability for oil and water of the materials can selectively separate or absorb oil ingredients from mixtures and fulfil the separation [18,19,20].

Carbon materials usually have satisfactory hydrophobicity and easily regenerative abilities, which are widely used as absorbents in oil removal materials [21]. Inagaki et al., fabricated carbon fibre oil-absorbing materials, which exhibited excellent heavy oil removal performance and could be easily regenerated by centrifugation and filtration [22,23]. Furthermore, carbon nanotubes (CNTs) with a three-dimensional network structure and excellent mechanical strength, compressibility, and thermal and electrical properties, have a wide range of application prospects in solid polymer membranes for fuel cells, catalyst carrier, super capacitor, sensor, and water treatment [24,25,26,27,28,29]. Considering the above performance, Fan et al. fabricated CNTs oil adsorption materials with excellent recovery property. It is reported that the aligned CNTs materials had high oil adsorption capacity and good recycling performance [30]. However, carbon nanotubes are rarely reported and fabricated as filtration oil–water separation materials, especially when dealing with oil–water emulsion mixtures.

**Scheme 1:**
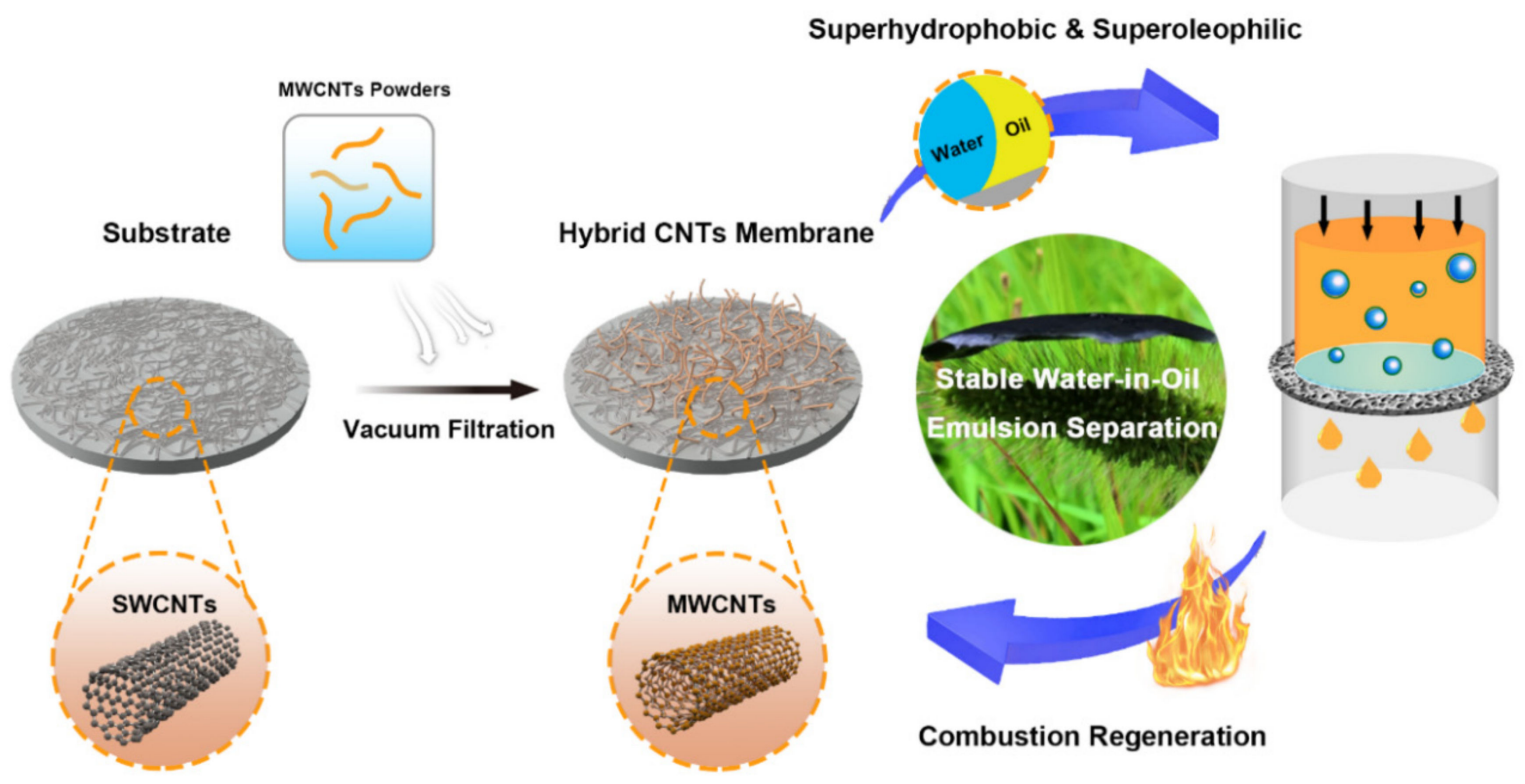
Schematic description of the preparation of the hybrid CNTs film, and the emulsions’ separation and combustion regeneration process of the films.

In this work, a novel ultralight superhydrophobic-superoleophilic CNTs film with double-layer structures was fabricated for oil–water emulsions separation as shown in Scheme 1. The films were made by two simple steps including vacuum filtration and annealing. It is found that the as-prepared CNTs films can accomplish separation of various types of water-in-oil emulsions at ambient temperature. In addition, the films can be easily regenerated through a rapid burning procedure. The CNTs films are stable, easily stored and produced. These findings demonstrate that the as-prepared CNTs films have great potential in practical applications such as wastewater treatment, oil purification and so forth.

## 2. Materials and Methods

### 2.1. Preparation of the Hybrid CNTs Film

The hybrid CNTs film was prepared by vacuum filtration method (Scheme 1). In general, MWCNTs powders were ultrasonically dispersed in ethanol with the concentration of 25 μg/mL, and the thickness of different concentrations is shown in Figure S1 (see details in Supplementary Materials). SWCNTs films were cut into small circles with a diameter of 50 mm and placed on a filter. Then the MWCNTs powders suspension was poured onto the SWCNTs film by vacuum filtration to form the MWCNT/SWCNT hybrid double-layer film. The hybrid film was dried at 150 °C for 0.5 h and then annealed at 700 °C in argon atmosphere, which was intended to remove hydrophilic groups. SWCNT films used here were purchased from Chuzhou Broad Creative Energy Technology LTD (Chuzhou, China). MWCNT powders were purchased from Cnano Technology Company (Zhenjiang, China), which synthesized via a fluidized bed method.

### 2.2. Water-in-Oil Emulsion Separation Experiments

Four types of surfactant-stabilized emulsions were prepared including water in chloroform, toluene, gasoline, and diesel emulsions. For each emulsion, 0.05 g Span 80 (Sinopharm Chemical Reagents, Beijing, China) was first added into 50 mL oil phase, then 1.0 mL water was injected in the solution. The mixtures were stirred at least for 1 h at room temperature before being used. It is worth noting that Span 80 was used as the surfactant.

The as-prepared CNTs film was fixed in the filtration device. Then, four kinds of the water-in-oil emulsions were poured onto the film, respectively, and the separation was driven by using a vacuum filtration system at about 0.1 Mpa. All emulsions could be successfully separated in one step. The oil purities of filtrates separated from the above emulsions were all greater than 99.3%, and three samples were measured to get the average value.

### 2.3. Characterizations and Instruments

Scanning electron microscopy images of the as-prepared films were obtained using a field emission scanning electron microscope (JSM-7500F, JEOL, Tokyo, Japan). Contact angle was measured on an OCA20 instrument (Data-Physics, Berlin, Germany) and the average value of five measurements was performed at different positions on the same sample. The X-ray photoelectron spectroscopy (XPS) was carried out on a VGESCALAB 220-IXL (Thermo Fisher, Shanghai, China) spectrometer using an Al Kα X-ray source (1486.6 eV). Loading capacity and mechanical properties were tested by a Materials Testing System (Instron 5943, a Division of Illinois Tool Works Inc, 825 University Avenue Norwood, MA, USA). The water content in the collected filtrates was acquired by a Karl Fischer Titrator (GR Scientific, Cou-Lo Aquamax KF Moisture Meter, Changsha, China). Optical microscopy images were taken on a Nikon ECLIPSE LV100POL (Shanghai, China) polarizing optical microscope.

## 3. Results and Discussions

### 3.1. Surface Morphology and Wetting Behaviors

**Figure 1:**
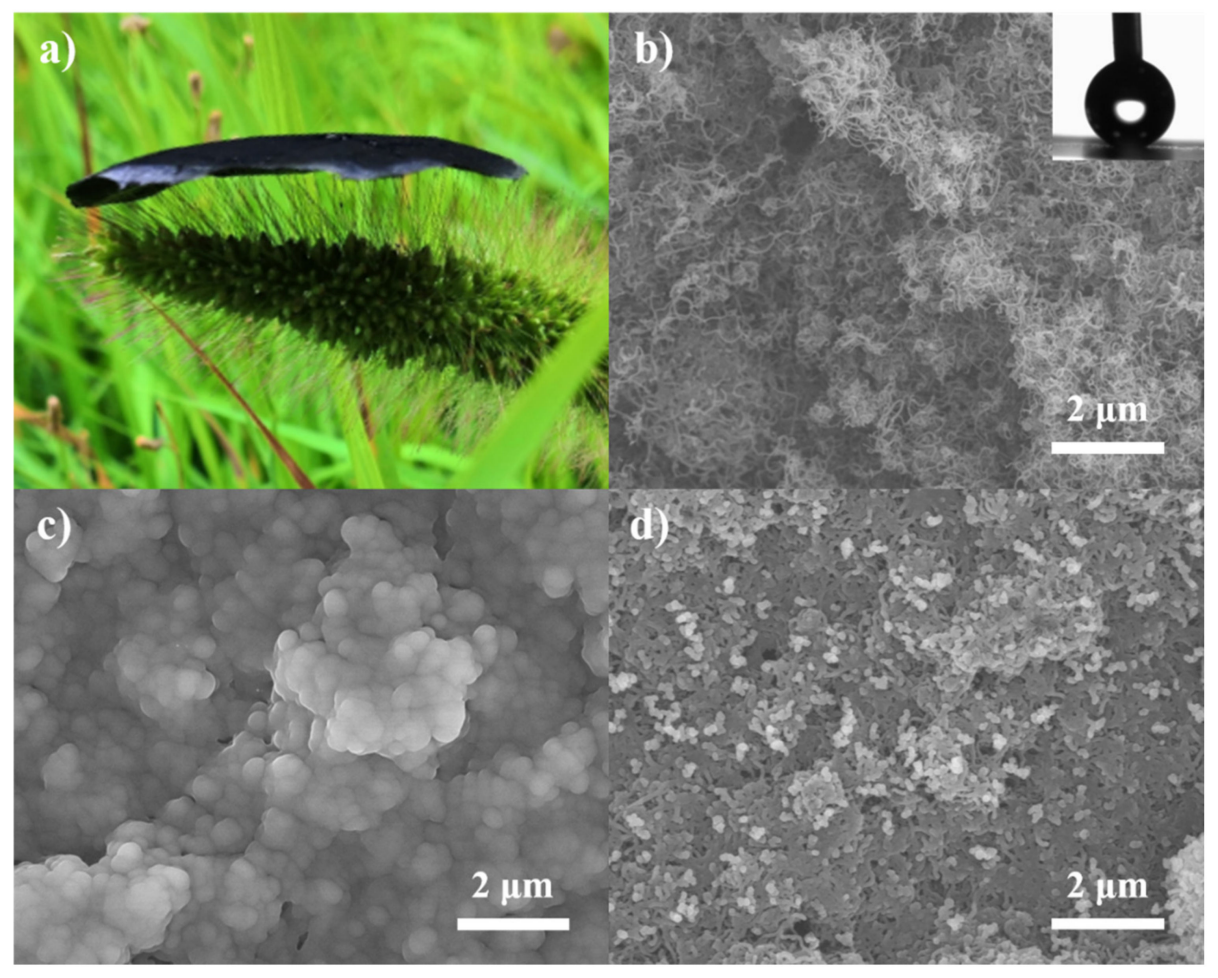
(**a**) Photograph of a piece of hybrid film standing on a green bristlegrass, proving the ultralight properties. (**b**) SEM image of the original CNTs film showing a porous structure in which nanowires are intertwined and overlapped with each other; the inset image is the photograph of the water droplet that stays on the film with water contact angle of about 159°. (**c**) SEM image of the films after using, which remains a large amount of oils residue. (**d**) SEM image of film after regeneration. Water and oil residue would volatilize and be removed by the film drying and the surfactant would remain on the film.

The hybrid CNTs film fabricated by the vacuum filtration method was ultralight. Figure 1a shows that one piece of the as-prepared film could stand freely on a green bristlegrass, proving the scalable possibility of preparing ultralight films. The scanning electron microscope (SEM) images shown in Figure 1b–d are the images of the original, used, and regenerated hybrid films. The as-prepared original film has numerous MWCNTs regularly distributed across the SWCNTs film (Figure 1b). After filtering four kinds of the water-in-oil emulsions, oil residue, water and surfactant remain on the surface of the film, and subsequent cleaning and burning remove most of the surfactant. A large amount of residue remains on the film as shown in Figure 1c, and the residue also blocked the porous structure. However, after one quick combustion regeneration process, most of the residues are removed and the subsequent uses about films are just as effective as the first use. (Figure 1d). The residual oils have been burned off; the porous structure of carbon nanotubes reappears on the surface, demonstrating the recyclability and stability of the films. The inset image in Figure 1b is the photograph of a water droplet that stays on the hybrid film, and the water contact angle (WCA) of the film is 156.9 ± 1.0°.

### 3.2. Combustion Regeneration Process

**Figure 2:**
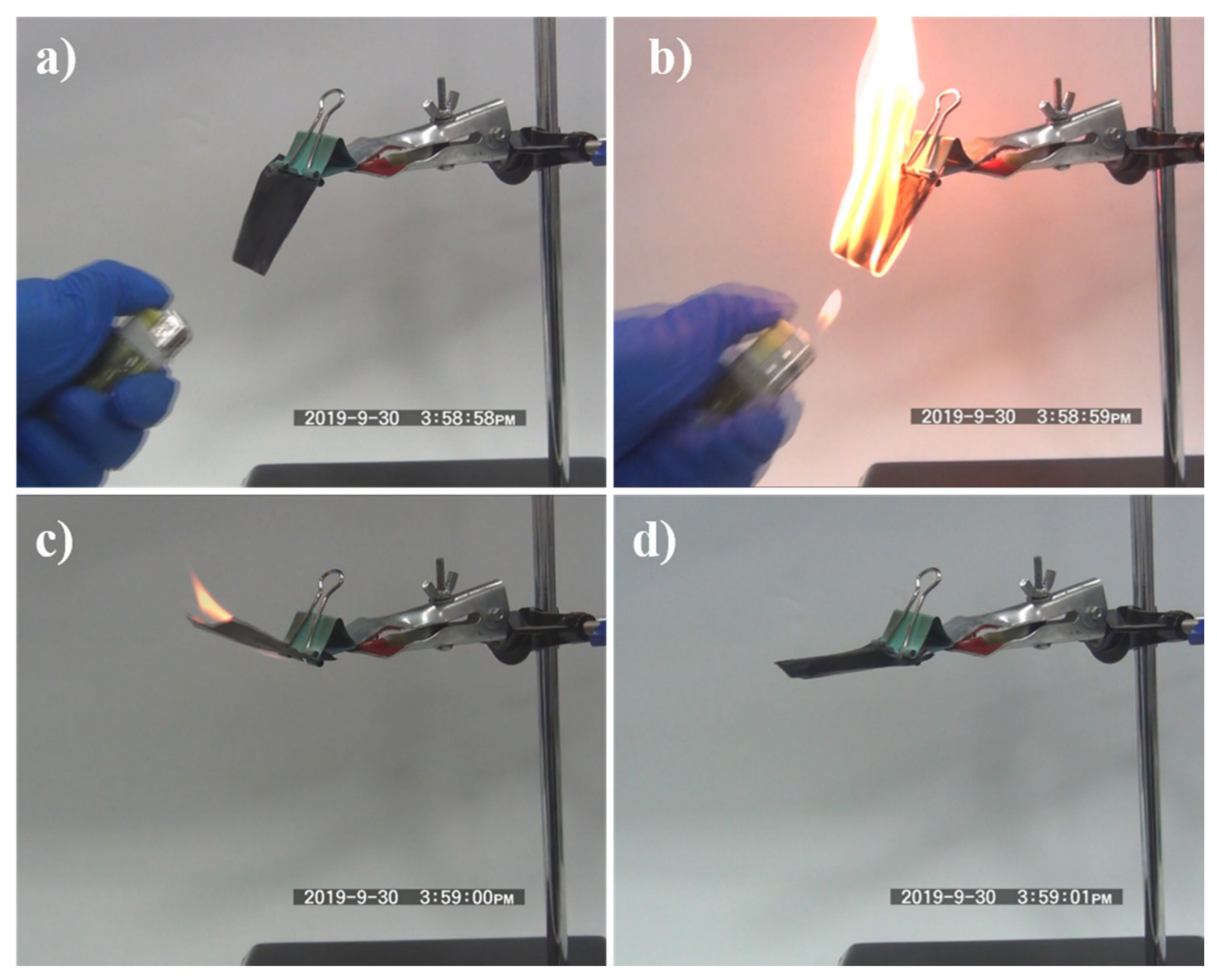
(**a**–**d**) The combustion regeneration process of the hybrid CNTs films after oil–water separation.

The regeneration of the hybrid CNTs film was through a simple combustion process. The process was recorded and some screen shots are presented in Figure 2. The used film with filtered emulsion residue has been fixed on the holder as shown in Figure 2a. After being ignited by a lighter, the oil residue on the film was burned easily. From Figure 2b–d, it can be seen that the complete burning lasted less than 2 s. Combined with the above SEM image in Figure 1d, most of the water and oil residue would volatilize and be removed by the film drying and the surfactant would remain on the film. The film can basically return to its original performance, indicating the rapid regeneration ability and stability of the hybrid films.

### 3.3. Surface Composition Characterization

**Figure 3:**
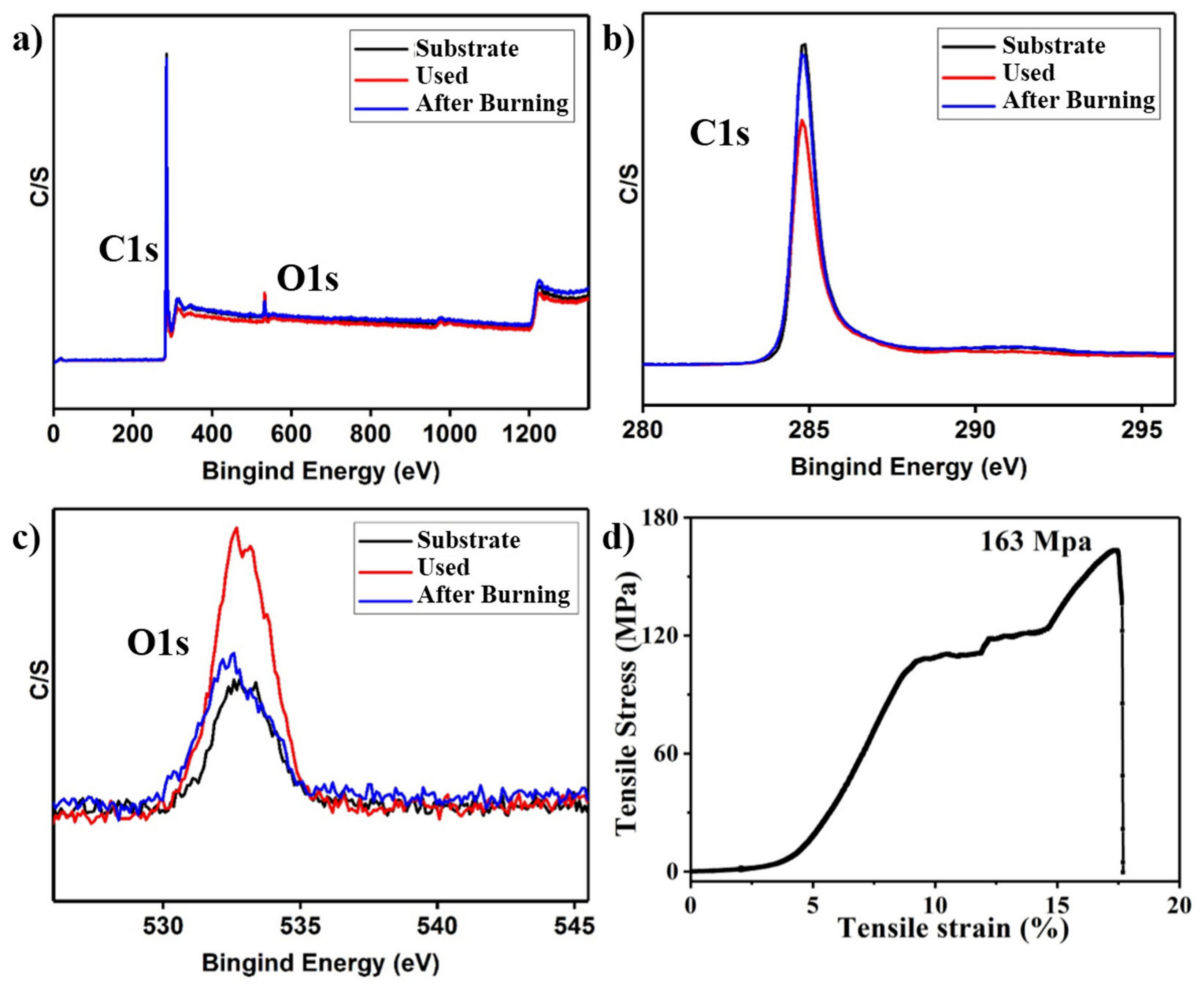
(**a**) The XPS survey spectra of the original film (black), used film (red) and regenerated film (blue). XPS spectra of C1s (**b**) and O1s (**c**). (**d**) Tensile stress curve of the CNTs film.

The composition change of the CNTs films before and after being used, and regenerated were examined by X-ray photoelectron spectroscopy (XPS), which is presented in Figure 3. Two basic elements including carbon and oxygen were surveyed by scanning bonding energy from 0 to 1200 eV. Two main peaks at 284.9 and 532.8 eV have been labelled in Figure 3a, which represent C1s and O1s. From Table S1, the C content on the original film was 98.97%, while it was 97.53% on the used film. After the quick burning process, the C content on the regenerated film returned to 98.71%. Figure 3b is the narrow scan spectra of C1s on the three films. It is visually shown that the peak of the used film was lower than the original hybrid CNTs film. While the peak of C1s on the spectra of regenerated film returns as before, it is worth noting that the black spectrum represents the original substrate, which is obscured by the blue spectrum. In the meantime, there were surfactants and oil residues on the surface of the film; oxygen existed in these residues, causing the O1s content to first increase after use for a couple of times. After combustion, the residues on the film would generate carbon dioxide and water, resulting in a decrease in oxygen content (as shown in Figure 3c). The contents of O1s of the original film, used film, and regenerated film were 0.78%, 2.06%, and 0.99%, respectively. There was a peak shift in the O1s spectrum to lower binding energy after burning due to the C=O existed in residues. Besides, burning might change Fe^2+^ to Fe_2_O_3_ or Fe_3_O_4_, causing a peak shift to lower binding energy. The XPS analysis further confirmed that the CNTs films can basically return to the original state after a quick burning process. Additionally, tensile test was performed to evaluate the mechanical property of the as-prepared hybrid CNTs films. In Figure 3d, the hybrid CNTs film has high tensile strength of about 163 Mpa, which is stronger than the nature films. High mechanical performance of the film is mainly due to the strong SWCNTs films after the vacuum filtration process, which ensures good practicability of the films in oil–water separation application.

### 3.4. Separation Efficiency

**Figure 4:**
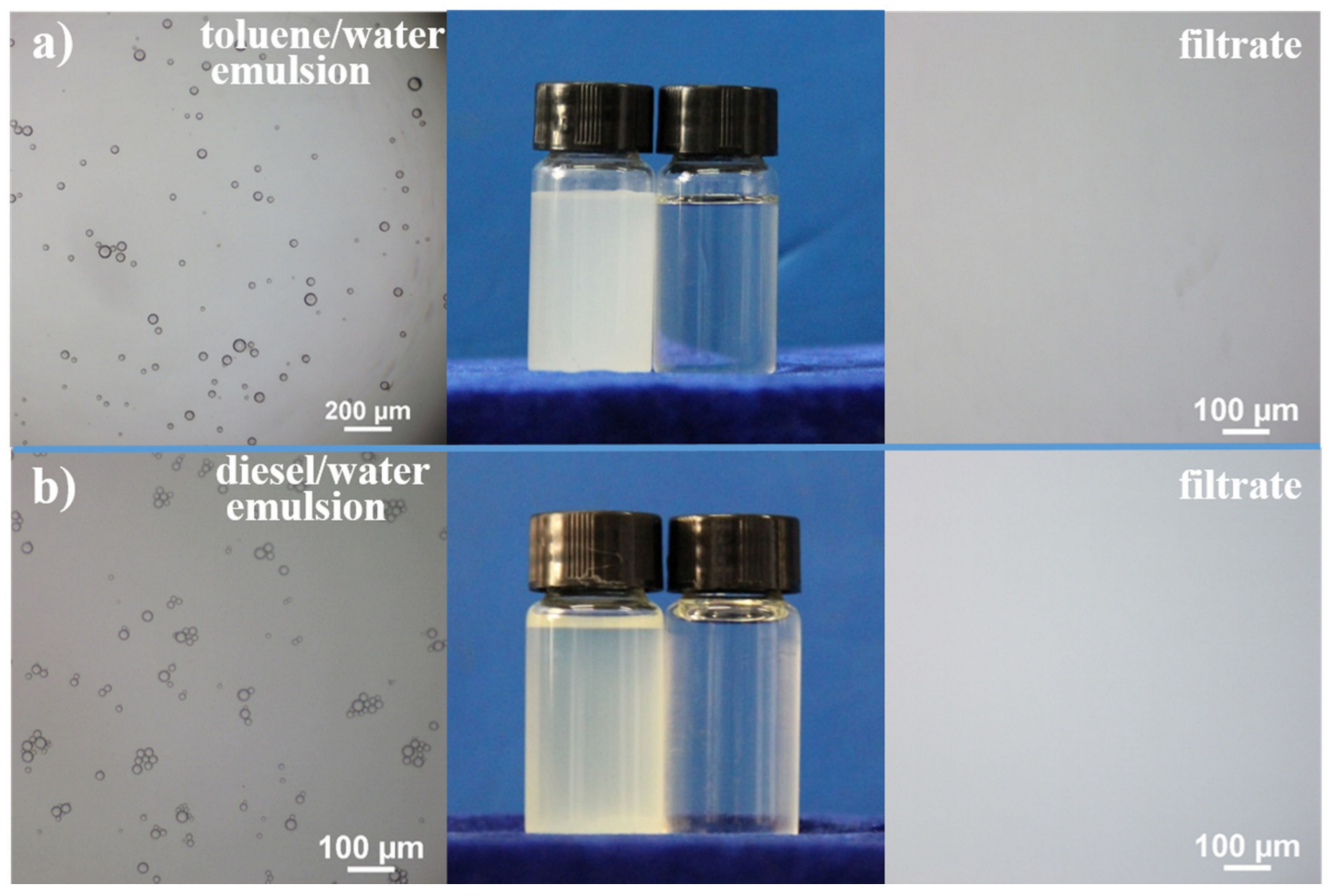
Optical microscope images of water-in-toluene emulsions (**a**) and water-in-diesel (**b**) before and after separation, and the photographs of Span-80 stabilized emulsions and filtrates in the middle.

A series of stabilized water-in-oil emulsions including water in chloroform, toluene, gasoline, and diesel have been prepared to test the separation capacity of the hybrid CNTs films. The water-in-oil emulsions contained about 1% of water and 1 mg/mL Span 80. The CNTs film was fixed into the separation apparatus. The as-prepared water-in-oil emulsions were poured onto the film and the separation was driven by using a vacuum filtration system at about 0.1 Mpa. The four kinds of oils pass through the film thoroughly and water from the demulsified emulsion was blocked and stayed on the upper side of the film. All emulsions could be successfully separated in one step with high efficiencies. Figure 4a,b shows the separation results of taking water-in-toluene and water-in-diesel emulsions as examples. The photographs of water-in-gasoline (chloroform) were presented in Supplementary Materials in Figure S2. Compared to the original milky white or light yellow feed emulsions, the collected filtrates were clear and transparent. Optical microscopy of emulsions and filtrates were also examined before and after separation. From the images, we can observe that the feed emulsions contained plenty of droplets before filtration while no single droplet was observed in the collected filtrates. The optical microscope images and photographs of emulsions and filtrates both confirmed the well removal of water during the separation process.

**Figure 5:**
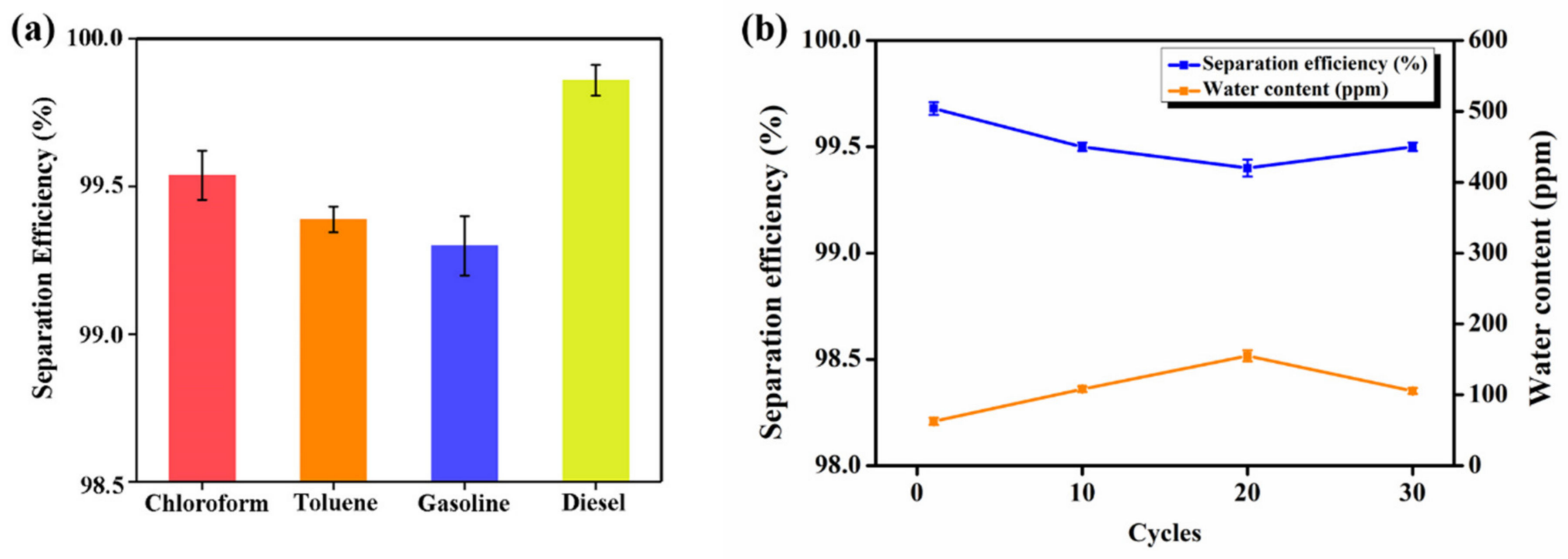
(**a**) Separation efficiencies of four kinds of Span-80 stabilized water-in-oil emulsions after one time separation by the hybrid CNTs films; (**b**) The recyclability tests of the hybrid CNTs films.

The separation efficiency was calculated by the water rejection coefficient (*R*(%)) according to:

(1) $$R(\%)=(1-C_{P}/C_{0})\times 100$$

where *C*_0_ and *C_p_* are the water concentration of the original emulsions and the filtrates. The water concentration of the filtrates after one time separation was examined by using a Karl Fischer Titrator. The water content in each filtrate was the average of three measurements with different samples. As shown in Figure 5a, the separation efficiencies of four kinds of Span-80 stabilized emulsions were higher than 99.3%, demonstrating the excellent separation ability of the hybrid CNTs films. After calculation, the quantity of oil that can be separated was about 2.5 mL/cm^2^ during one time separation. Additionally, the recyclability of the material was also tested as shown in Figure 5b. After 30 times of separation, the efficiency is still above 99.3% and water content below 150 ppm, which demonstrates the good recyclability of the films. The photograph of water-in-toluene emulsion and filtrates was presented in Supplementary Materials in Figure S3.

### 3.5. Chemical and Mechanical Stability

**Figure 6:**
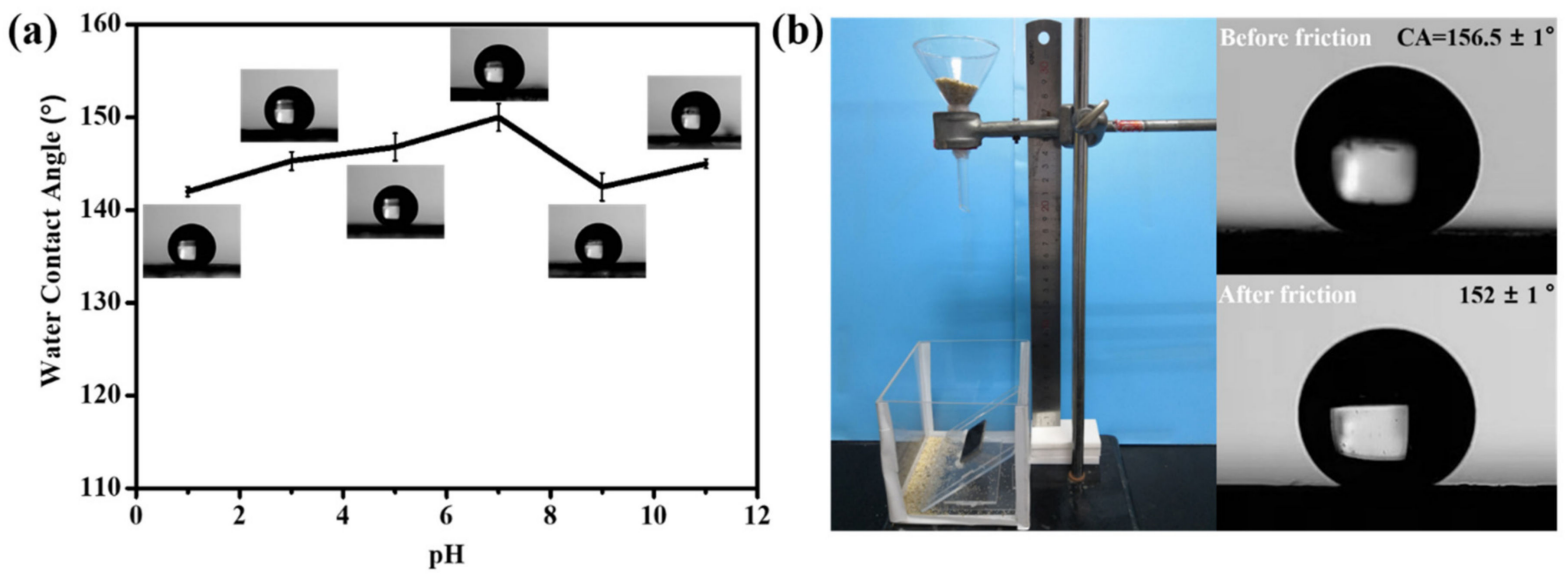
(**a**) Chemical stability test of the film after immersing in the solutions with different pH values: The water contact angles stay greater than 140°; (**b**) Mechanical stability test of the film withstands the friction by sand: The water contact angle stays greater than 152°.

The chemical stability of the composite membrane was evaluated by measuring the water contact angles at different pH. Figure 6a shows the water contact angles in ambient temperature of the membrane after being immersed in different pH solutions for 30 min. It can be seen that the film remains hydrophobic with greater than 140° water contact angle for 1 ≤ pH ≤ 11. In addition, the mechanical stability of the membrane was tested by measuring the water contact angles before and after friction by sand. Sand fell off for 20 cm and the weight was 100 g. After friction, the water contact angles remained above 152°. So the film exhibits excellent stability under chemical corrosion and mechanical friction.

## 4. Conclusions

In conclusion, we have developed a novel hybrid CNTs film with superhydrophobic and superoleophilic property. The as-prepared films can separate various surfactant stabilized-water-in-oil emulsions with high separation efficiency. Moreover, the hybrid CNTs film is ultralight and can be easily regenerated by rapid combustion process. In addition, they exhibit excellent chemical stability under acidic and alkaline conditions and friction tests. The hybrid film is easily fabricated, stored and reused. Therefore, the functional CNTs films have a forward outlook for use in practical applications such as oil purification, treating wastewater in industry and daily life.

## Supplementary Materials

The following are available online at https://www.mdpi.com/2077-0375/11/1/1/s1, Figure S1. Thickness of MWCNTs on SWCNT films with different concentrates: (**a**) SWCNT film (13.93 μm); (**b**) 20 µg/mL (5.881 μm); (**c**) 25 µg/mL (6.118 μm); (**d**) 30 µg/mL (8.040 μm). The inset images are the photographs of the water droplets stay on the film, Figure S2. The comparison of photograph of the water-in-chloroform (gasoline) emulsions and filtrates after separation, Table S1. Element contents of the original film, used film, and regenerated film based on XPS analysis, Figure S3. The comparison of photograph of the water-in-toluene emulsions and filtrates after separation.

## References

[1] Zhang Q., Cao Y., Liu N., Zhang W., Chen Y., Lin X., Tao L., Wei Y., Feng L. (2016). A facile approach for fabricating dual-function membrane: Simultaneously removing oil from water and adsorbing water-soluble proteins. Adv. Mater. Interfaces.

[2] Liu N., Zhang M., Zhang W., Cao Y., Chen Y., Lin X., Xu L., Feng L., Li C., Wei Y. (2015). Ultralight free-standing reduced graphene oxide membranes for oil-in-water emulsions separation. J. Mater. Chem. A.

[3] Li H., Li Y., Liu Q. (2013). ZnO nanorod array-coated mesh film for the separation of water and oil. Nanoscale Res. Lett..

[4] Cao Y., Chen Y., Liu N., Lin X., Feng L., Wei Y. (2014). Mussel-inspired chemistry and Stöber method for highly stabilized water-in-oil emulsions separation. J. Mater. Chem. A.

[5] Zhang W., Liu N., Cao Y., Chen Y., Xu L., Lin X., Feng L. (2015). A solvothermal route decorated on different substrates: Controllable separation of an oil/water mixture to a stabilized nanoscale emulsion. Adv. Mater..

[6] Zhang W., Liu N., Cao Y., Lin X., Liu Y., Feng L. (2017). Superwetting porous materials for wastewater treatment: From immiscible oil/water mixture to emulsion separation. Adv. Mater. Interfaces.

[7] Guha I.F., Varanasi K.K. (2018). Separating nanoscale emulsions: Progress and challenges to date. Curr. Opin. Colloid Interface Sci..

[8] Chen C., Weng D., Mahmood A., Chen S., Wang J. (2019). The separation mechanism and construction of surfaces with special wettability for oil/water separation. ACS Appl. Mater. Interfaces.

[9] Sato M., Sumita I. (2007). Experiments on gravitational phase separation of binary immiscible fluids. J. Fluid Mech..

[10] Megid M.H., Amer A.A., Elsayed K.H. (2014). Coagulation and dissolved air floatation for treatment of oilwater emulsion. Int. J. Eng. Sci..

[11] Chen G. (2004). Electrochemical technologies in wastewater treatment. Sep. Purif. Technol..

[12] Kwon W.T., Park K., Han S.D., Yoon S.M., Kim J.Y., Bae W., Rhee Y.W. (2010). Investigation of water separation from water-in-oil emulsion using electric field. J. Ind. Eng. Chem..

[13] Rajasekhar T., Trinadh M., Veera Babu P., Sainath A.V., Reddy A.V. (2015). Oil–water emulsion separation using ultrafiltration membranes based on novel blends of poly(vinylidene fluoride) and amphiphilic tri-block copolymer containing carboxylic acid functional group. J. Membr. Sci..

[14] Zhu Y., Wang D., Jiang L., Jin J. (2014). Recent progress in developing advanced membranes for emulsified oil/water separation. NPG Asia Mater..

[15] Xue Z., Cao Y., Liu N., Feng L., Jiang L. (2014). Special wettable materials for oil/water separation. J. Mater. Chem. A.

[16] Yang S., Si Y., Fu Q., Hong F., Yu J., Al-Deyab S.S., EI-Newehy M., Ding B. (2014). Superwetting hierarchical porous silica nanofibrous membranes for oil/water microemulsion separation. Nanoscale.

[17] Zhang G., Li M., Zhang B., Huang Y., Su Z. (2014). A switchable mesh for on-demand oil–water separation. J. Mater. Chem. A.

[18] Yong J., Huo J., Chen F., Yang Q., Huo X. (2018). Oil/water separation based on the natural materials with super-wettability: Recent advances. Phys. Chem. Chem. Phys..

[19] Wei Y., Qi H., Gong X., Zhao S. (2018). Specially wettable membranes for oil-water separation. Adv. Mater. Interfaces.

[20] Ge M., Cao C., Huang J., Zhang X., Tang Y., Zhou X., Zhang K., Chen Z., Lai Y. (2018). Rational design of materials interface at nanoscale towards intelligent oil-water separation. Nanoscale.

[21] Hu Y., Liu X., Zou J., Gu T., Chai W., Li H. (2013). Graphite/isobutylene-isoprene rubber highly porous cryogels as new sorbents for oil spills and organic liquids. ACS Appl. Mater. Interfaces.

[22] Inagaki M., Kawahara A., Nishi Y., Iwashita N. (2002). Heavy oil sorption and recovery by using carbon fiber felts. Carbon.

[23] Inagaki M., Kawahara A., Konno H. (2002). Sorption and recovery of heavy oils using carbonized fir fibers and recycling. Carbon.

[24] Rambabu G., Bhat S.D. (2014). Simultaneous tuning of methanol crossover and ionic conductivity of sPEEK membrane electrolyte by incorporation of PSSA functionalized MWCNTs: A comparative study in DMFCs. Chem. Eng. J..

[25] De Volder M.F., Tawfick S.H., Baughman R.H., Hart A.J. (2013). Carbon nanotubes: Present and future commercial applications. Science.

[26] Cao Q., Rogers J.A. (2009). Ultrathin films of single-walled carbon nanotubes for electronics and sensors: A review of fundamental and applied aspects. Adv. Mater..

[27] Hu L., Hecht D.S., Gruner G. (2010). Carbon nanotube thin films: Fabrication, properties, and application. Chem. Rev..

[28] Schnorr J.M., Swager T.M. (2010). Emerging applications of carbon nanotubes. Chem. Mater..

[29] Liu Y.H., Yi B., Shao Z.G., Xing D.M., Zhang H.M. (2006). Carbon Nanotubes Reinforced Nafion Composite Membrane for Fuel Cell Applications. Electrochem. Solid State Lett..

[30] Fan Z., Yan J., Ning G., Wei T., Qian W., Zhang S., Zheng C., Zhang Q., Wei F. (2010). Oil sorption and recovery by using vertically aligned carbon nanotubes. Carbon.

