# Supplementary material for: Ultralight, Strong and Renewable Hybrid Carbon Nanotubes Film for Oil-Water Emulsions Separation
Source: Membranes (Basel). 2020 Dec 22;11(1):1. doi: 10.3390/membranes11010001 (PMC7821942; doi:10.3390/membranes11010001)
Supplement: Supplementary file 1 [file membranes-11-00001-s001.pdf]

membranes

Supplementary Information

Ultralight, Strong and Renewable Hybrid Carbon Nanotubes Film for Oil/Water Emulsions Separation

Yamei Lu [1,2], Yingze Cao [2], Yi Jia [2], Chunai Dai [1,*] and Pengfei Wang [2,*]

[1] Department of Chemistry, School of Science Beijing Jiaotong University, Beijing 100044, China; 18126243@bjtu.edu.cn

[2] Qian Xuesen Laboratory of Space Technology, China Academy of Space Technology, Beijing 100094, China; caoyingze@qxslab.cn (Y.C.); jiayi@qxslab.cn (Y.J.)

* Correspondence: chadai@bjtu.edu.cn (C.D.); wangpengfei@qxslab.cn (P.W.)

Received: 3 November 2020; Accepted: 15 December 2020; Published: date

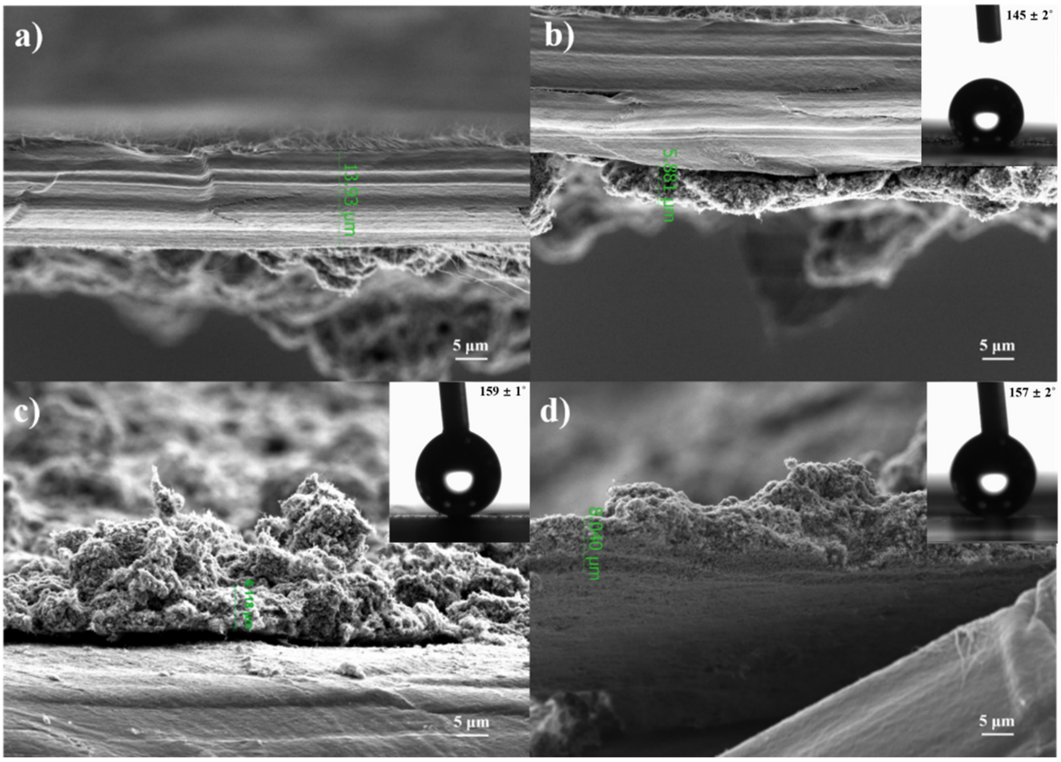

Figure 1. Thickness of MWCNTs on SWCNT films with different concentrates: (**a**) SWCNT film (13.93 μm); (**b**) 20 μg/mL (5.881 μm); (**c**) 25 μg/mL (6.118 μm); (**d**) 30 μg/mL (8.040 μm). The inset images are the photographs of the water droplets stay on the film.

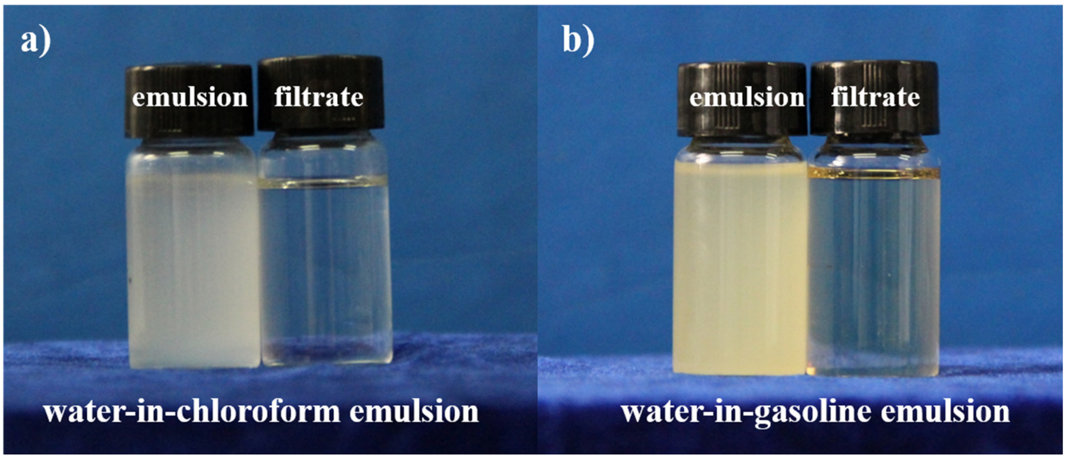

Figure 2. The comparison of photograph of the (**a**) water-in-chloroform and (**b**) water-in-gasoline emulsions and filtrates after separation.

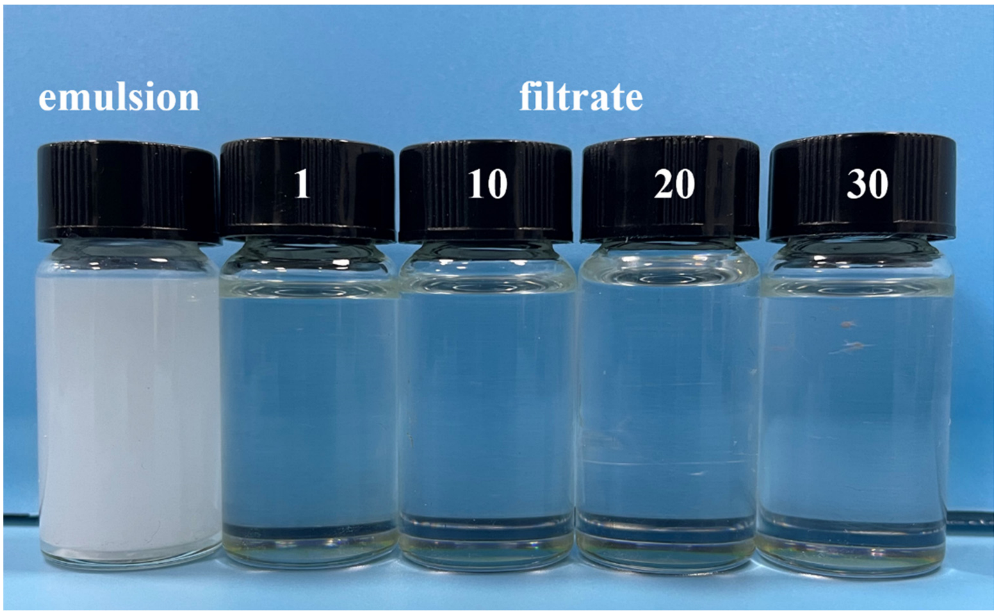

Figure 3. The comparison of photograph of the water-in-toluene emulsions and filtrates after separation.

Table 1. Element contents of the original film, used film, and regenerated film based on XPS analysis.

	Original Film	Used Film	Regenerated Film
C1s	98.94	97.53	98.71
O1s	0.78	2.06	0.99
N1s	0.23	0.36	0.26
Fe2p	0.05	0.05	0.04

Note: a small amount of N mainly from air and Fe was due to the use $C_{10}H_{10}Fe$ (Ferrocene) as the catalyst in the preparation of MWCNT powders.
